# MiR-425 Promotes Migration and Invasion in Bladder Cancer by Targeting Dickkopf 3

**DOI:** 10.7150/jca.40233

**Published:** 2020-03-13

**Authors:** Jin-zhuo Ning, Wei-min Yu, Fan Cheng, Ting Rao, Yuan Ruan

**Affiliations:** Department of Urology, Renmin Hospital of Wuhan University, Wuhan 430060, Hubei Province, P.R.China

**Keywords:** miR-425, DKK-3, bladder cancer, migration, invasion

## Abstract

**Background:** Bladder cancer (BC) is a common malignancy with high morbidity and mortality. MicroRNAs (miRNAs) are critical post-transcriptional regulators in various cancers. This study aimed to investigate the effect of miR-425 on the migration and invasion of BC.

**Methods:** The expression of miR-425 and Dickkopf 3 (DKK3) was examined in clinical BC specimens. T24 and 5637 BC cell lines were employed and transfected with miR-425 inhibitors. The correlation between miR-425 and DKK3 was determined by a luciferase reporter assay. Cell migration and invasion capacity were measured by wound healing and Transwell assays. The expression levels of DKK3, E-cadherin, N-cadherin and vimentin were analysed by Western blotting and qRT-PCR.

**Results:** miR-425 was negatively correlated with the expression of DKK3 in clinical BC specimens. Further studies identified DKK-3 as a direct target of miR-425. Moreover, knockdown of miR-425 promoted the expression of DKK3 and suppressed cell migration and invasion capacity. miR-425 silencing increased E-cadherin levels but decreased vimentin and N-cadherin protein levels in T24 and 5637 cells.

**Conclusion:** Our study indicated that miR-425 promoted the migration and invasion of BC via targeting DKK3.

## Introduction

Bladder cancer (BC) is a common malignant tumour worldwide, with an estimated 380,000 new cases and 15,000 deaths annually 1. In recent years, the incidence and mortality of BC has been increasing rapidly 2. BC is often characterised by a high tumour recurrence rate and a high invasive and migratory ability, resulting in an unsatisfactory prognosis 3. Therefore, exploring the mechanism underlying the progression of BC will be helpful in development of more effective molecular biomarkers.

Dickkopf-related protein 3 (DKK3) is the primary member of the human Dickkopf family that functions by encoding secreted proteins in determining cell fate during embryonic development 4. Dkk-3 is involved in various cellular activities and is widely expressed in brain, heart, lungs, liver, colon and kidneys 5,6. Notably, it has been observed that reducing and/or silencing Dkk3 is closely related to a broad range of cancer cell types, including BC 7,8. However, Tsujimura *et al* 9 demonstrated that the expression of Dkk-3 was exceptionally increased in BC. These findings collectively indicated that DKK3 plays a critical role in the progression of BC.

MicroRNAs (miRNAs) are a series of endogenous, single-stranded non-coding RNAs, 20-24 nucleotides in length. They are capable of negatively regulating the expression of genes by binding to their 3'-untranslated-regions 10. Accumulated evidence has suggested that miRNAs are tightly associated with numerous biological processes, including differentiation, carcinogenesis and morphogenesis, and aberrant expression of miRNAs contributed to tumour migration and invasion in tumour genesis 11-13. For example, miR-495 promoted bladder cancer cell growth and invasion by repressing PTEN 14, miR-129 regulated metastasis of prostate cancer by binding to CP110 15, miR-33 was down-regulated in breast cancer and acted as a tumour suppressor through modulating the HMGA2 gene 16. However, the biological effects of miR-425 in the progression of BC and its underlying mechanisms have not been described.

In the current study, we analysed the discrepant expression between miR-425 and DKK3 in BC tumour tissues, then predicted and identified that DKK3 was a direct downstream target of miR-425. Furthermore, we confirmed the regulatory effect of miR-425 on the migration and invasion of BC cells. These results suggested that miR-425 may play a critical role in the progression of BC and could be used as a novel therapeutic strategy for BC.

## Materials and Methods

### Clinical specimens

The present study was approved by the Animal Experimental Ethics Committee of Wuhan University (Wuhan, China) and carried out according to the guidelines of ethical management. A total of 32 BC specimens and paired normal tissues were selected from the Department of Urology, Ren'min Hospital of Wuhan University during 2017-2019. Patients signed consent forms and provided relevant clinical information. The stages of all samples were determined according to tumour, node, and metastasis (TNM) staging system of the American Joint Committee on Cancer (AJCC) classification system. All tissues were divided into two parts, with one half fixed in 4% paraformaldehyde. The other half was immediately frozen and stored at -80°C for later analysis. The clinical data are shown in Table 1.

### Cell culture and transfection

Human bladder epithelial cells (SV-HUC-1) and Bladder cancer cell lines (T24 and 5637) were purchased from ATCC (American Type Culture Collection, Manassas, VA, USA) and maintained in RPMI 1640 medium (Gibco, Gaithersburg, MD, USA) supplemented with 10% foetal bovine serum (FBS) at 37°C under normoxic conditions (5% CO_2_, 95% O_2_). Cells were transfected with miR-425 inhibitor and negative control inhibitor (NC inhibitor) (Biossci, Wuhan, China) using Lipofectamine 2000 (Invitrogen, Carlsbad, CA, USA) according to the manufacturer's protocol. Cells were treated by 48 h starvation for further analyses.

### Plasmid Construction and Luciferase Reporter Assays

The putative and mutated miR-425 target binding sequences in DKK3 were synthesised and cloned into a luciferase reporter to generate wild-type (DKK3-WT) or mutated-type (DKK3-MUT) reporter plasmids. The mutant 3'UTR sequence of DKK3 was obtained using an overlap-extension PCR method. Then, the sequences including the predicted wild and mutant target sites were subcloned into a psiCHECK-2 vector (Promega Corporation, Madison, WI). For the luciferase reporter assay, T24 cells were seeded into 24-well plates and were co-transfected with miR-425 inhibitor or NC inhibitor using Lipofectamine 2000. Cells were harvested at 48 h post-transfection. Luciferase activities were measured using a Dual-Luciferase Reporter Assay System (Promega, Madison, WI, USA).

### Immunohistochemistry

The expression of DKK3 was measured by immunohistochemical staining. Briefly, tissues were fixed in 4% paraformaldehyde, embedded in paraffin and then cut in 4-μm sections. Subsequently, the sections were incubated with rabbit polyclonal anti-Dkk3 (ab187532; Abcam, Cambridge, UK) antibodies at 4°C overnight. After three washes with PBS, all sections were incubated with goat anti-rabbit IgG for 30 min, stained with diaminobenzidine (DAB) reagents and observed using an Olympus BX50 light microscope (Olympus Corporation, Tokyo, Japan).

### Quantitative real-time PCR

Total RNA was extracted from clinical specimens and BC cells using TRIzol Reagent (Invitrogen Life Technologies, Carlsbad, CA, USA), and the RNA purity was determined using a DU800 UV/Vis Spectrophotometer (Beckman Coulter, CA, USA). Subsequently, total cellular RNAs were reversed transcribed into cDNA using a reverse transcription reagent kit (Toyobo, Osaka, Japan). Real-time quantitative PCR was performed via an Applied Biosystems SYBR Green Mix Kit and an ABI 7900 Real-Time PCR System (Applied Biosystems Life Technologies, Foster City, CA, USA). The expression of miR-425 and DKK3 mRNA was normalised to U6 (for miRNAs) and GAPDH (for mRNAs), respectively. The relative amount of miRNA or mRNA was calculated using the 2^-∆∆Ct^ method and the primer sequences used are shown in Table 2.

### Western blot analysis

Total cellular proteins were extracted with RIPA buffer with PMSF (Beyotime, Beijing, China). Protein concentration was qualified using a bicinchoninic acid assay kit (BCA; Beyotime, Beijing, China). Briefly, equivalent amounts of protein were separated by 10% sodium dodecyl sulphate-polyacrylamide gels electrophoresis (SDS-PAGE) and were subsequently transferred to polyvinylidene fluoride (PVDF) membranes followed by blocking and incubation with primary antibodies against DKK3 (ab187532; Abcam, Cambridge, UK), E-cadherin (ab1416; Abcam, Cambridge, UK), N-cadherin (ab18203; Abcam, Cambridge, UK) and vimentin (ab92547; Abcam, Cambridge, UK) at 4°C overnight. After incubation with goat anti-rabbit IgG anti-body at room temperature for 1 hour, all bands were measured using an enhanced chemiluminescence (ECL) system kit (MultiSciences, Hangzhou, China).

### Wound healing assay

Cells were seeded in 6-well plates and were cultured in complete medium for 12 hours. The wounds were generated with 100 μL tips in the confluent cell layers. The wounds were observed and the gap distances of migrating cells was measured at 0 and 24 h using a microscope (Olympus, Japan).

### Cell Invasion and Migration Assays

The migratory and invasive ability of T24 and 5637 cells were evaluated using Transwell chambers (Corning LifeSciences). 1×10^5^ cells were seeded in the upper Matrigel-coated chamber, and medium containing 10% FBS was placed in the lower chamber. After incubation for 24 h, cells in the upper chamber membrane were removed. Then, cells on the lower chamber membrane were fixed and stained with 0.1% crystal violet for 30 minutes.

### Statistical analysis

All data were presented as the means ± SD. Differences were assessed by two-tailed Student's t test and χ^2^ test as appropriate. A value of P<0.05 was considered statistically significant. All experiments were performed at least 3 times. Statistical analyses were performed using SPSS 20.0 (SPSS, Chicago, IL).

## Results

### Enhanced expression of miR-425 correlates with down-regulation of DKK3 expression in BC samples

**Figure 1:**
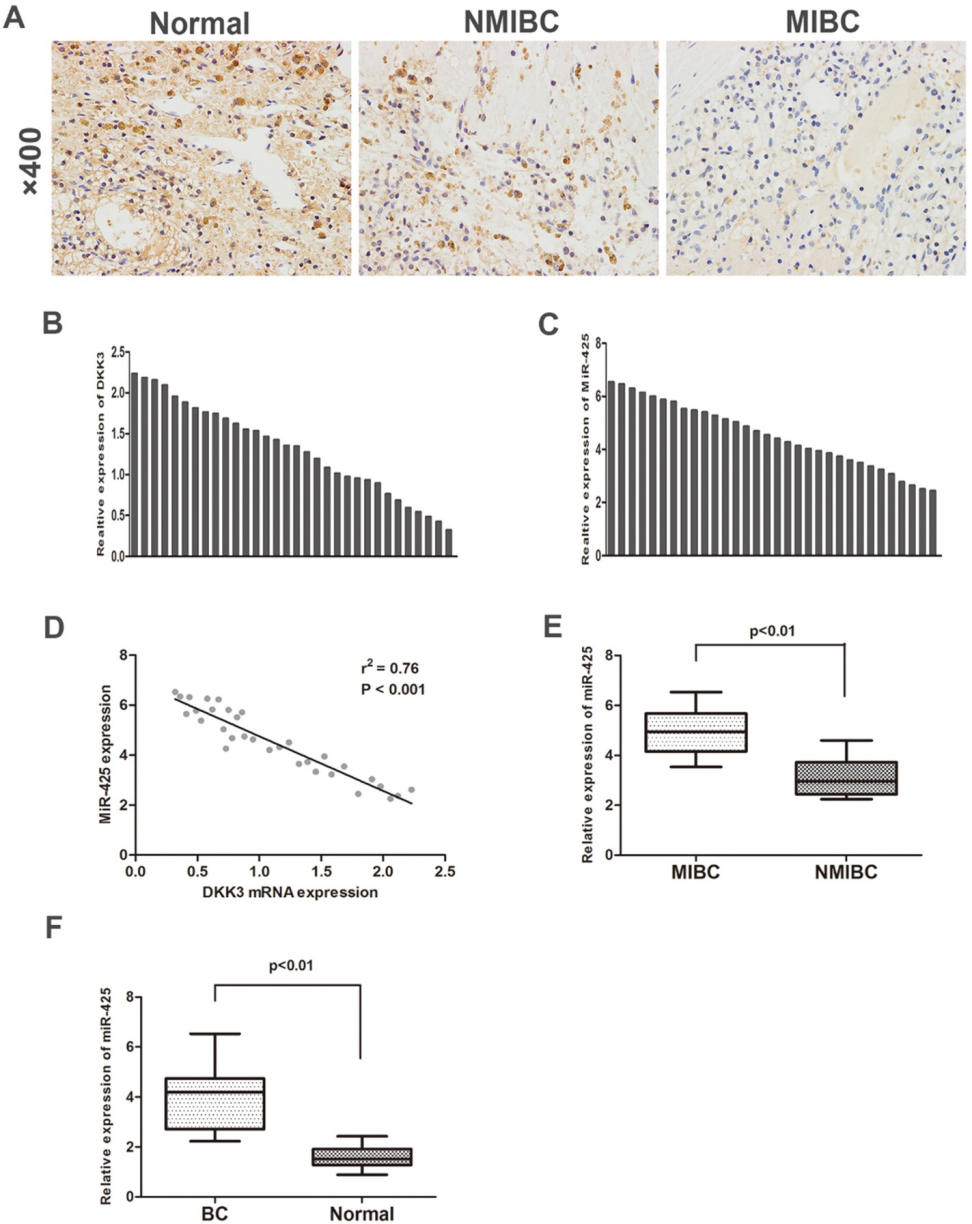
** Enhanced expression of miR-425 correlates with downregulation of DKK3 expression in BC samples. (A)** Immunohistochemical staining of DKK3 in normal bladder tissues, NMIBC tissues and MIBC tissues. **(B,C)** QRT-PCR analysis of DKK3 and miR-425 mRNA expression in human BC tissues from 32 patients. **(D)** A two-tailed Pearson's correlation analysis reveals that the mRNA expression of miR-425 is inversely correlated with the expression of DKK3. **(E)** Box plots represent relative expression of miR-425 in NMIBC tissues and MIBC tissues. **(F)** Box plots represent relative expression of miR-425 in BC tissues and adjacent normal tissues.

To investigate the expression of DKK3 in BC samples and adjacent normal control samples, we first analysed 32 cases of BC tissues specimens and paired normal tissues by immunohistochemistry staining and qRT-PCR. The expression of DKK3 was significantly lower in BC tissues than in adjacent normal tissues. Additionally, compared with BC is the most significant malignancy of the urinary system. Statistically, approximately 80% of the diagnosed tumours are non-muscle invasive bladder cancer (NMIBC) tissues, muscle invasive bladder cancer (MIBC) tissues exhibited significantly lower expression of DKK3 (Fig. 1 A,B). Nonetheless, PCR results revealed that miR-425 expression dramatically increased during the progression of BC (Fig. 1 C,E,F). Two-tailed Pearson's correlation 'analysis was performed, and the result demonstrated that the expression of miR-425 was negatively correlated with DKK3 expression (r^2^=0.76, P<0.01) (Fig. 1D).

### miR-425 directly targets DKK3 and negatively regulates DKK3 expression

**Figure 2:**
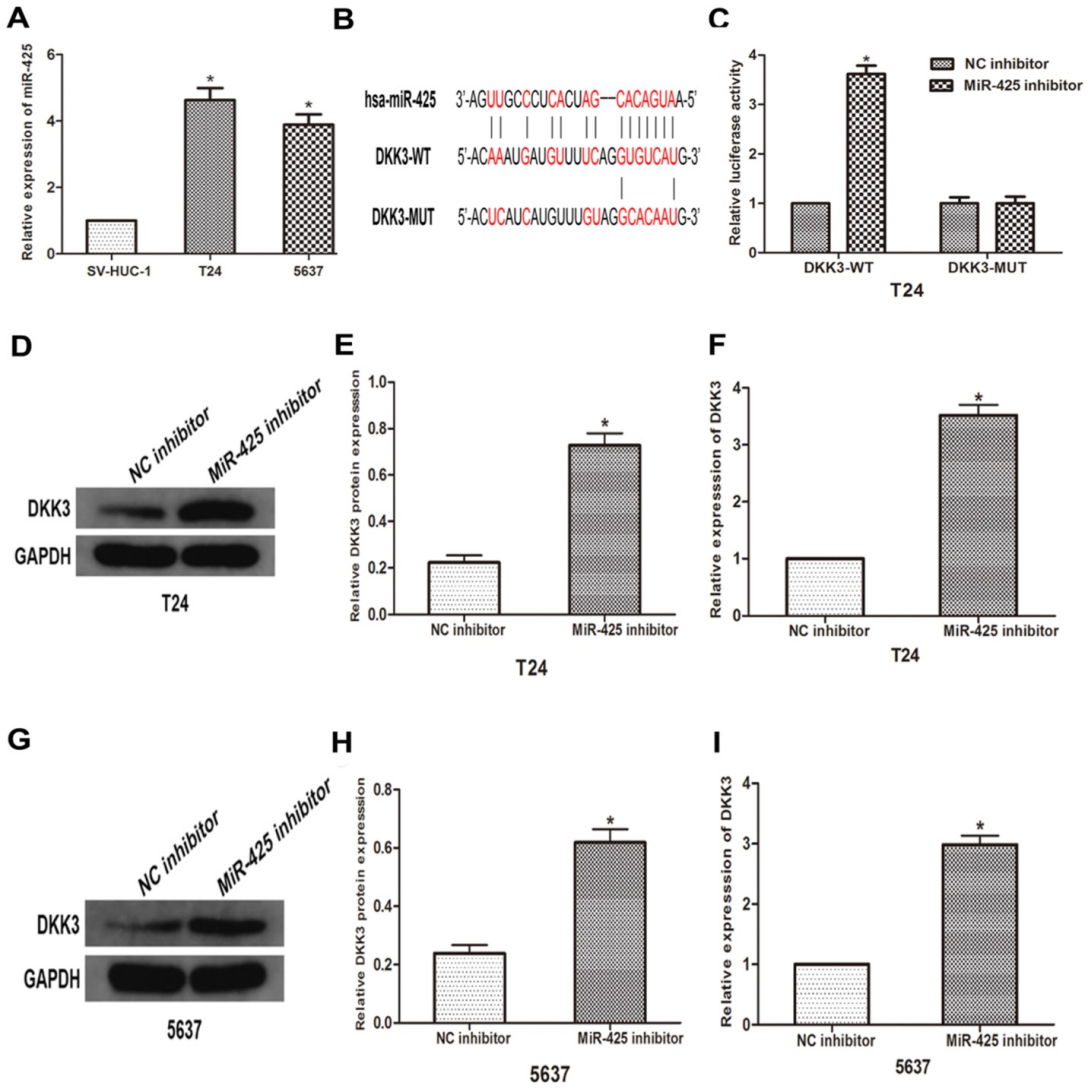
** MiR-425 directly targets DKK3 and negatively regulates DKK3 expression. (A)** The expression of miR-425 in BC cell lines (T24 and 5637) and human bladder epithelial cells (SV-HUC-1) was examined by qRT-PCR. *P<0.05 vs.SV-HUC-1 group. **(B)** Sequence alignment of predicted miR-425 binding sites within the DKK3 3'UTR and its mutated sequence for luciferase reporter assay. **(C)** Luciferase reporter assay was performed in T24 cells that were co-transfected with miR-425 inhibitor and reporter vectors containing DKK3 3'UTR or mutated DKK3 3'UTR. Relative luciferase activities are presented. **(D-I)** Western blot and qRT-PCR analyses of DKK3 expression after transfection with miR-425 inhibitor in T24 and 5637 cells. (*p < 0.05 vs. NC inhibitor)

To further explore the relationship between miR-425 and DKK3, we first examined the expression of miR-425 in BC cell lines (T24 and 5637) and human bladder epithelial cells (SV-HUC-1). As the result of qRT-PCR showed that miR-425 was up-regulated in varying degrees in BC cells compared with normal cells (Fig 2A). we predicted that miR-425 was an upstream regulator of DKK3 by using open access software (Targetscan, miRanda and miRwalk2.0), and a putative binding site for miR-425 was identified within the 3'UTR (Fig. 2B). To confirm this prediction, we cloned a luciferase reporter sequence in the 3'UTR of DKK3, which contains the putative miR-425 binding sites. A mutant reporter vector of the 3'UTR of DKK3 containing luciferase reporter was used as a negative control. Data from luciferase reporter assay revealed that knockdown of miR-425 significantly increased reporter vector activity of DKK-3 3'UTR in T24 cells but had no effect on the mutated reporter vector (Fig. 2C). Subsequently, we transfected T24 and 5637 cells with miR-425 inhibitor. Western blot and qRT-PCR analyses showed that miR-425 silencing led to an increase in DKK3 expression in T24 and 5637 BC cells (Fig. 2 D-I). Collectively, these results suggested that miR-425 can negatively regulate the expression of DKK3 by directly targeting it.

### miR-425 promotes cell migration and invasion of BC cells

**Figure 3:**
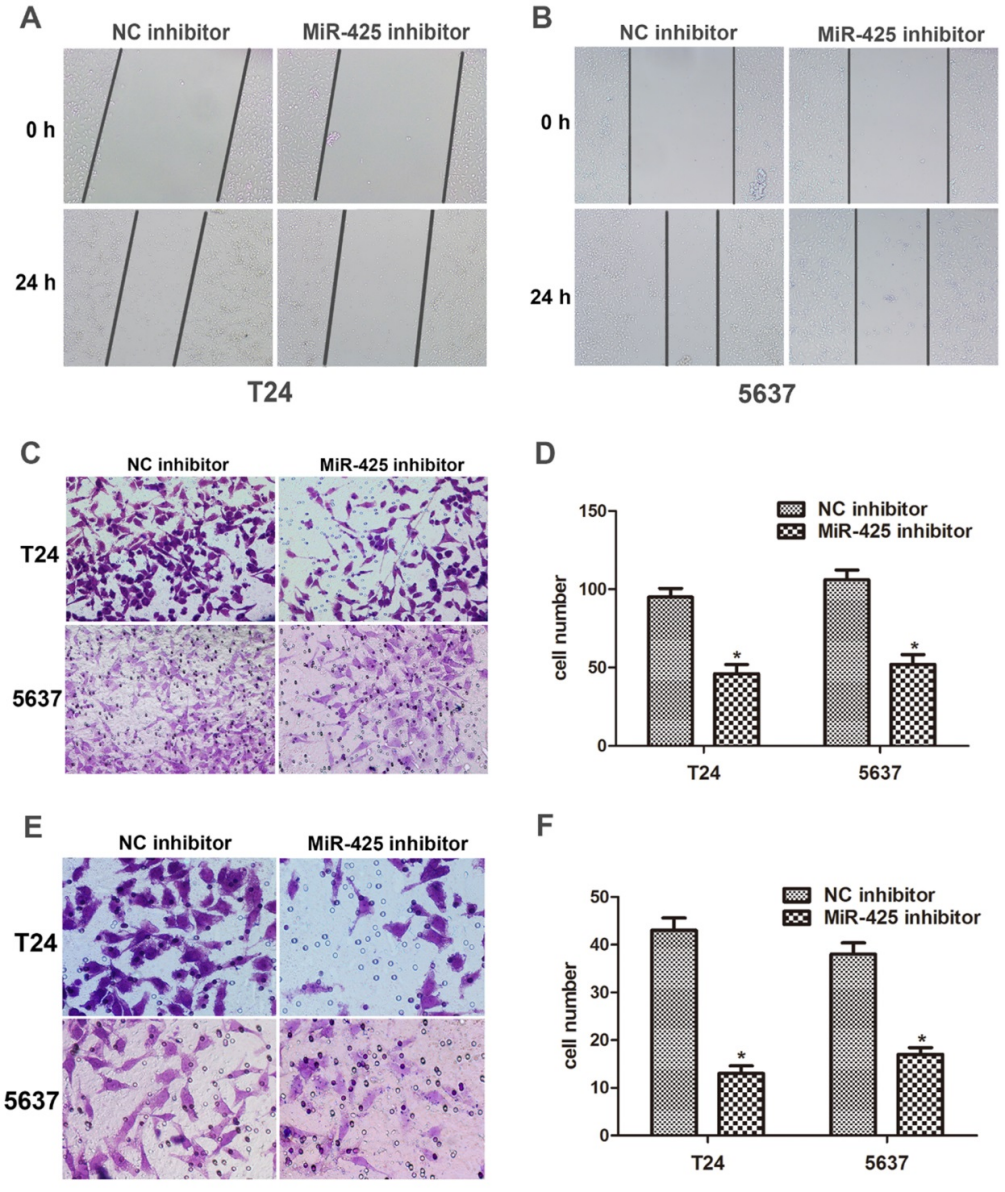
** MiR-425 promotes cell migration and invasion of BC cells. (A, B)** Wound healing assay was performed to evaluate cell migratory ability in T24 and 5637 cells transfected with miR-425 inhibitor at 0 and 24 hours. **(C,D)** Transwell migration assay was performed to investigate the migratory ability after transfection with miR-425 inhibitor in T24 and 5637 cells. **(E,F)** Transwell invasion assay with Matrigel-coated membranes was performed to show the invasive ability after transfection with miR-425 inhibitor in T24 and 5637 cells. (*p < 0.05 vs. NC inhibitor)

We next examined the effect of miR-425 on the migration and invasion in BC cells. The data of the wound healing assay showed that knockdown of miR-425 remarkably reduced cell migration in T24 and 5637 cells (Fig. 3 A,B). By using a Transwell assay, we found that down-regulation of miR-425 inhibited cell migration in T24 and 5637 cells (Fig. 3 C,D). Compared with NC inhibitor, the number of invaded cells was substantially lower when cells were treated with miR-425 inhibitor (Fig. 3 E,F). Taken together, these findings indicated that miR-425 promoted cell migration and invasion in BC cells.

### Effects of miR-425 on epithelial-mesenchymal transition (EMT) in BC cells

**Figure 4:**
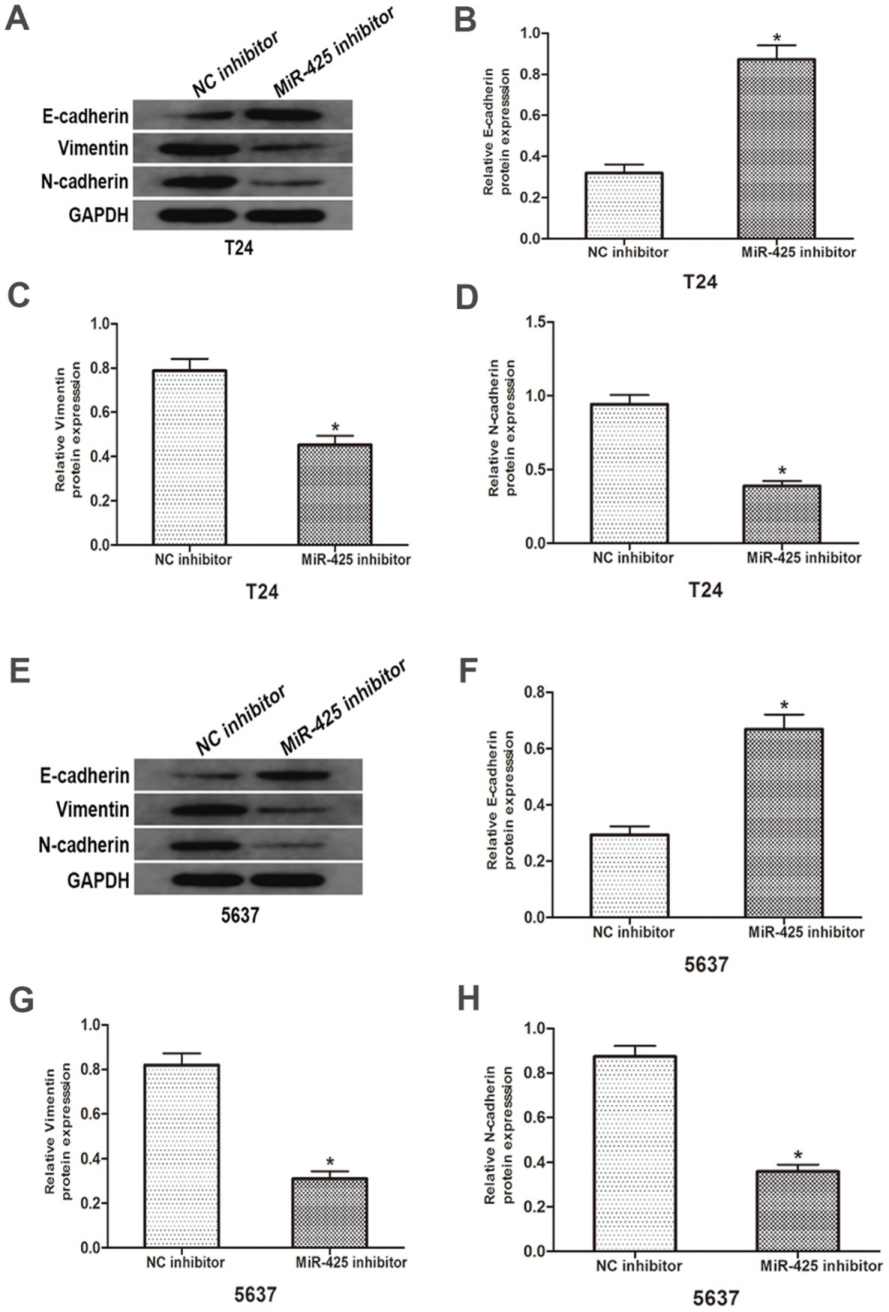
** Effects of miR-425 on Epithelial-mesenchymal transition (EMT) in BC cells.** E-cadherin, N-cadherin and vimentin protein levels of T24 and 5637 cells after transfection with miR-425 inhibitor were detected by western blot analysis. GAPDH was used as an internal control. (*p < 0.05 vs. NC inhibitor)

To investigate the influence of miR-425 in the EMT progression of BC, we transfected T24 and 5637 cells with miR-425 inhibitor. As shown in Fig. 4, Western blot revealed that down-regulation of miR-425 increased E-cadherin expression but lowered vimentin and N-cadherin protein levels in T24 and 5637 cells. Taken together, these results demonstrated that miR-425 may be an important regulator in the EMT progression of BC.

## Discussion

BC is the most significant malignancy of the urinary system. Statistically, approximately 80% of the diagnosed tumours are NMIBC 17. The majority of NMIBC can be detected and treated in the early stages that have a 5-year survival rate of more than 90% 18,19. However, 50-70% of NMIBC are likely to recur, and 10-20% of cases may rapidly progress to MIBC^20^,^21^. For MIBC, its 5-year survival may decrease lower than 50%, which drops off to 20% in the case of metastatic disease 22. Hence, it is critical to understand the molecular interactions in the progression of BC and provide new prognostic treatments as well. Previous studies have demonstrated that miRNAs are closely related to tumour migration and invasion in cancer cells by targeting genetic markers. Based on these results, we tried to identify that miRNAs that regulate the progression of BC.

Previous studies have demonstrated that miRNAs regulate gene expression post-transcriptionally, and abnormally expressed miRNAs regulated RNA networks in tumour progression 23,24. It has been reported that miR-425 promoted invasion and metastasis in hepatocellular carcinoma via modulation of SCAI, while it inhibited melanoma metastasis by negatively regulating IGF-1 through repression of PI3K-Akt pathway 25,26. Furthermore, a recent study suggested that miR-425 regulated tumour progression by directly targeting PDCD10 in colorectal cancer 27. However, the biological function and clinical significance of miR-425 in the progression of BC remains unclear. Here, in the current study, we found a negative relevant relationship between miR-425 and DKK3 in BC specimens. Compared with normal adjacent tissues, BC tissues showed significant up-regulation of miR-425 whereas and down-regulation of DKK3 during the progression of the disease. Subsequently, we identified DKK3 as a direct downstream target of miR-425 using a luciferase reporter assay. These data provided evidence that miR-425 negatively regulated the expression of DKK3 in BC cells, in agreement with the observations from clinical samples.

DKK3 has been reported to be abnormally expressed in various cancers, contributing to migration and invasion of cancer cells through multiple pathways 28-30. For example, DKK3 suppressed tumour progression by regulating the B-catenin/EMT signalling pathway in pancreatic cancer Bxpc-3 cells 31. Moreover, knockdown of Dkk-3 inhibited cell migration and invasion in oral squamous cell carcinoma, possibly contributing to the activation of Wnt signalling pathways 32. In our *in vitro* studies, we employed T24 and 5637 cells to simulate the progression of BC. Our data showed that miR-425 knockdown inhibited cell migration and invasion in BC cell lines. Our results were consistent with those of previous reports, suggesting that miR-425 acts on an oncogene and has a potential as a novel biomarker for BC.

Tumour metastasis is the main cause of death in patients with malignant tumours, a multistep process in which tumour cells move from their primary site and form secondary tumours at distant anatomic sites 33. EMT is a primary process that contributes to the escape of metastatic cancer cells from the primary tumour, as characterised by down-regulation of E-cadherin expression and up-regulation mesenchymal markers (including N-cadherin and vimentin) 11. As our data revealed, knockdown of miR-425 significantly increased the expression of E-cadherin while decreasing the expression of N-cadherin and vimentin, suggesting that miR-425-mediated inhibition of DKK3 was associated with EMT in the progression of BC.

## Conclusions

Taken together, our data indicated that miR-425 functioned as an oncogene in BC cells by promoting cell migration and invasion capacity, and such biological effect may, at least in part, be attributed to the negative regulation of DKK3. These findings suggest that miR-425 could be used as a diagnostic biomarker for BC treatment.

## Figures and Tables

**Table 1:** Relative DKK3 expression and the clinical characteristics of 32 patients with BC

		DKK3 expression			
Variable	Group	Low	High	Total	P value
Sex	Male	11	6	17	0.304
	Female	7	8	15	
Age	<60	9	11	20	0.515
	≥60	4	8	12	
Tumor grade	NMIBC	3	17	20	<0.001
	MIBC	9	3	12	
Tumor size	<3cm	4	13	17	0.018
	≥3cm	11	4	15	

**Table 2:** RT-PCR primer sequences

Gene	Primer sequences (5'-3')
**DKK3**	F: CTGTGTGTCTGGGGTCACTG
	R: GCTCTAGCTCCCAGGTGATG
**miR-425**	F: AAUGACACGAUCACUCCCGUUGA
	R: CCAGUGCUCGACUCAUCGCGGCG
**U6 snRNA**	F: CTCGCTTCGGCAGCACATATACT
	R: ACGCTTCACGAATTTGCGTGTC
**GAPDH**	F: ACAGCAACAGGGTGGTGGAC
	R: TTTGAGGGTGCAGCGAACTT
